# Influence of Conditional Cash Transfers on the Uptake of Maternal and Child Health Services in Nigeria: Insights From a Mixed-Methods Study

**DOI:** 10.3389/fpubh.2021.670534

**Published:** 2021-07-06

**Authors:** Uchenna Ezenwaka, Ana Manzano, Chioma Onyedinma, Pamela Ogbozor, Uju Agbawodikeizu, Enyi Etiaba, Tim Ensor, Obinna Onwujekwe, Bassey Ebenso, Benjamin Uzochukwu, Tolib Mirzoev

**Affiliations:** ^1^Health Policy Research Group, College of Medicine, University of Nigeria Enugu, Nsukka, Nigeria; ^2^Department of Health Administration and Management, Faculty of Health Sciences and Technology, University of Nigeria Enugu, Nsukka, Nigeria; ^3^School of Sociology and Social Policy, University of Leeds, Leeds, United Kingdom; ^4^Centre for International Health and Development, University of Leeds, Leeds, United Kingdom; ^5^Department of Global Health and Development, London School of Hygiene and Tropical Medicine, London, United Kingdom

**Keywords:** conditional cash transfer, maternal and child health, Nigeria, financial incentives, unintended consequences, utilization

## Abstract

**Background:** Increasing access to maternal and child health (MCH) services is crucial to achieving universal health coverage (UHC) among pregnant women and children under-five (CU5). The Nigerian government between 2012 and 2015 implemented an innovative MCH programme to reduce maternal and CU5 mortality by reducing financial barriers of access to essential health services. The study explores how the implementation of a financial incentive through conditional cash transfer (CCT) influenced the uptake of MCH services in the programme.

**Methods:** The study used a descriptive exploratory approach in Anambra state, southeast Nigeria. Data was collected through qualitative [in-depth interviews (IDIs), focus group discussions (FGDs)] and quantitative (service utilization data pre- and post-programme) methods. Twenty-six IDIs were conducted with respondents who were purposively selected to include frontline health workers (*n* = 13), National and State policymakers and programme managers (*n* = 13). A total of sixteen FGDs were conducted with service users and their family members, village health workers, and ward development committee members from four rural communities. We drew majorly upon Skinner's reinforcement theory which focuses on human behavior in our interpretation of the influence of CCT in the uptake of MCH services. Manual content analysis was used in data analysis to pull together core themes running through the entire data set.

**Results:** The CCTs contributed to increasing facility attendance and utilization of MCH services by reducing the financial barrier to accessing healthcare among pregnant women. However, there were unintended consequences of CCT which included a reduction in birth spacing intervals, and a reduction of trust in the health system when the CCT was suddenly withdrawn by the government.

**Conclusion:** CCT improved the utilization of MCH, but the sudden withdrawal of the CCT led to the opposite effect because people were discouraged due to lack of trust in government to keep using the MCH services. Understanding the intended and unintended outcomes of CCT will help to build sustainable structures in policy designs to mitigate sudden programme withdrawal and its subsequent effects on target beneficiaries and the health system at large.

## Introduction

Globally there has been an increasing effort to improve Maternal and Child Health (MCH), yet maternal mortality is still relatively high particularly in Sub-Saharan Africa (SSA). The vast majority of these preventable deaths (94%) occurred in low-resource settings (1).

In Nigeria, maternal mortality fell from 576/100,000 and under-five mortality of 128/1,000 live births in 2013 (2) to 512/100,000 and 132/1,000 live births in 2018, respectively (2). However, the use of ante-natal care (ANC) in Nigeria rose from 61% in 2013 to 67% in 2018, and skilled birth attendants from 38 to 43.3%, respectively (2). Furthermore, there are inequities in the utilization of MCH services, which have persisted over the years in Nigeria. The Nigerian Demography Health Survey found that women in urban communities are much more likely to receive ANC from a skilled provider compared to women in rural communities; 84 vs. 56%, respectively (2). Similarly, among children under-five (CU5) 157/1,000 live births occur in rural communities and 92/1,000 live births in urban communities (2).

Several economic, socio-cultural behavioral, and health system factors contribute to low levels and inequities in the utilization of MCH services. These include high direct and indirect costs of healthcare-seeking; the opportunity cost of being away from work or income-generating activities (3, 4); lack of transportation fare; and inaccessibility of health facilities due to high cost, attitude of health workers (5); asymmetry of information etc. (3). Also, the most common method of paying for health care in Nigeria is through out-of-pocket payments as over 69% of total health expenditure is incurred by households out-of-pocket at health facilities (6). Hence, low-income people are unable to afford health services (7) and geographical isolation of hard-to-reach communities from health facilities limits access to services; and to trained health workers (8, 9).

In order to improve service utilization and reduce inequities in access to health services financial incentives such as conditional cash transfers (CCTs) have been increasingly utilized among the important tools to encourage behaviors or actions to a desired one, such as providing and utilizing maternal and child health services (10). Financial incentives operate by providing an immediate reward for a behavior that will lead to long term health improvements (11). The incentives also influence health system actors on the demand side (to access the right services at the right time) and on the supply side (to provide the right services, of high quality, to all those who need them) (12).

Financial incentives particularly the CCTs are increasingly becoming popular in African countries including Nigeria which aid in improving health services (13–15), other sectors (16, 17) and poverty alleviation (18). The incentives also improve employee's performance (10), optimize the behavior of healthcare providers and their clients for maternal and neonatal health (12).

CCT in healthcare programmes functions by providing financial incentives to its users (specific population) to promote health-seeking behavior and create a positive impact on their health (11). CCTs have been suggested to potentially tackle financial barriers and motivational barriers to care-seeking and service utilization among beneficiaries (19, 20). CCTs seems to be particularly beneficial to poorer households, on the condition that those households will meet certain criteria such as periodic growth monitoring, up-to-date vaccinations, focused ANC, facility delivery, etc. to a health facility (18, 21, 22). While acknowledging the positive outcome of CCTs on health, of which has been the focus of most studies in recent times, scholars have called for greater attention to studying unintended consequences and moral concerns related to CCTs arising in a variety of local contexts (11, 23–25). The designs of CCT can be broadly similar but the specific designs are very different with regards to incentive size, time, duration etc. and these factors can make a difference (26).

In Nigeria, CCT was implemented within a special MCH intervention programme, called the Subsidy Reinvestment and Empowerment Programme (SURE-P) Maternal and Child Programme (SURE-P/ MCH) between 2012 and 2015 to improve uptake of MCH service in rural communities (27). Although a study has been published about CCTs in SURE-P/MCH (28), our paper provides in-depth exploration of how CCT worked, and the intended and unintended consequences of CCT implementation. This study therefore, aims to provide an insight into how CCT influenced MCH service uptake among pregnant women in rural Nigeria.

## Description of SURE-P Programme in Nigeria

**Figure 1:**
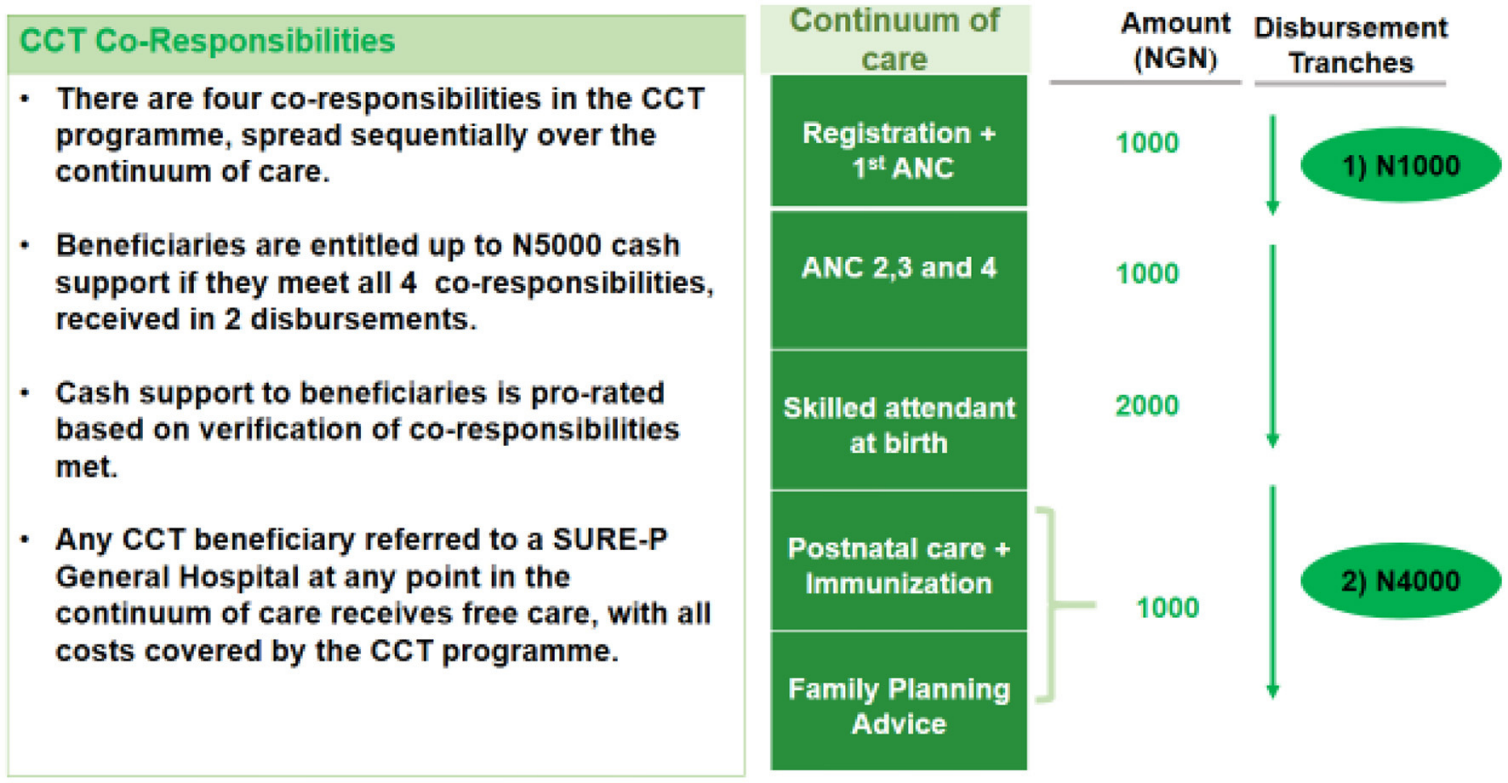
Method of CCT payment. Source: MCH Programme Implementation Unit (PIU). The SURE-P MCH Conditional Cash Transfer Programme is designed to encourage women to register and complete the continuum of care for MCH services. By the end of 2013, ~14,500 women had enrolled for the CCT Pilot Programme in eight states and the Federal Capital Territory (FCT).

In recognition of the poor MCH indices, the Nigerian Government between 2012 and 2015, implemented a Subsidy Reinvestment and Empowerment Programme (SURE-P), to invest profits from fuel revenues into a social protection fund for vulnerable populations (27). The SURE-P had a mother and child health (MCH) component (SURE-P/MCH) aimed at improving the lives of mothers and their infants. The SURE-P/MCH programme comprised both supply and demand components. The supply-side component aimed to broaden access to quality maternity services and improve MCH outcomes through providing resources: recruiting and training PHC workers [2,000 midwives, 10,000 community health extension workers (CHEWs)], infrastructural development, and the increasing availability of supplies and medicines. The demand component aimed to increase the utilization of health services during pregnancy and at birth using a conditional cash transfer (CCT) programme as a resource. Its target was to incentivize pregnant women to prioritize access and utilization of facility-based MCH services in rural communities by paying the women a stipend of 5,000 naira (about US$30) through the Continuum of pregnancy (Figure 1). All pregnant women in the community were eligible to participate in the programme irrespective of social-economic status because the CCT was not based on poverty reduction unlike other social security programmes but to support women in rural areas who are susceptible to financial hardship when accessing care. The programme was carried out in three clusters in each state, but, CCT was done only in one cluster in the nine States as a pilot intervention of which Anambra was among (28).

## Methods

### Study Area

This study was undertaken in Anambra State, southeast Nigeria. Anambra state was chosen as a case study for in-depth understanding of the inquiry into the CCT component of SURE-P/MCH programme. The state has a population of about 4.1 million and has a mix of urban and rural areas. MCH services are primarily provided from the Primary Health Centres (PHCs), each of which covers a given catchment population. In the context of the SURE-P/MCH programme, four PHCs are linked to a named general hospital for referral of Emergency Obstetric Complications (EOCs), and this is referred to as a cluster (4 PHCs +1 General hospital). Four PHC implemented the CCT programme in the State. However, there are some trained (maternity homes) and untrained (TBAs, Patent medicine Vendors) services who also offer unmonitored MCH provision.

The CCT intervention was originally designed to be administered using the above design as outlined in Figure 1. In practice, during implementation, beneficiaries only received money after their attendance of each service has been logged and verified, sometimes the length of this verification process meant that they were paid lump sums at the end of the delivery (28).

### Study Design

This was a 5-year multi-phased mixed-methods retrospective study in which various components of the intervention were evaluated to provide a conceptual information on the process (including barriers and enablers) that underlie the effect of CCTs on the uptake of health services. Qualitative data collection and secondary quantitative analysis of routinely collected administrative and CCT data were used to enable adequate triangulation and an in-depth and rich description of findings. CCT is a fairly new initiative in the country at the time and was initiated as a pilot intervention (28). The full methodology protocol is explained in detail elsewhere (29). This paper focuses one component (CCT intervention) of the wider study which was carried out in four (4) out of the 12 PHCs that implemented the programme.

Qualitative data [document reviews, in-depth interviews (IDIs) and focus group discussions] and quantitative methods [service utilization data from the Health Information Management System (HMIS)] were collected to cover pre-, during and post-SURE P/MCH programme. All the data collection tools/guides (Additional file 1) were developed by the researchers for the purpose of the study. Document reviews of relevant contextual literature were carried out. A logic map (30), of the expected process of programme interventions and how these could lead to outcomes was also developed. In-depth interviews of relevant stakeholders on both demand and supply-side were conducted to explore various dimensions of the CCT component of this programme and stakeholders' experiences in implementing CCT in the selected health facilities.

### Sampling and Data Collection

The SURE-P/MCH intervention was carried out in three health facility clusters, all three had the MCH interventions and only one had an additional CCT intervention. For this study, we purposively selected the SURE-P/MCH + CCT cluster within the larger project which is the focus of our study.

The respondents were purposively recruited from these facilities to include the facility managers (*n* = 4) and other health workers (Midwives and CHEWs) (*n* = 9). On the demand side, we also conducted four FGDs (6-8 participants per group) with service users (pregnant women who had received/were receiving maternal care services at the time of the study), and four FGDs with Village Health Workers (VHWs) (4-6 participants per group). The VHWs who were members of the communities were responsible for identifying pregnant women in their community and encouraging them to attend and use health care facilities. VHWs also assisted the women in registering for antenatal care (ANC) and enrolment into the CCT registers. We also had four FGDs with the family members of service users (6-8 participants per group), and four FGDs with the ward development committee (WDC) members (5-10 participants per group), who are community representatives that oversee the functioning of the facilities. National and State-level policymakers and programme managers (*n* = 13), who were either involved in the design or administration of the CCT intervention were also interviewed. These groups of respondents were interviewed by experienced researchers about their experiences of the CCT intervention from their various perspectives. Each interview was audio recorded, and handwritten notes were taken. Interviews and discussion were conducted at places convenient for the respondents/participants including health facilities, offices, village halls, and houses. Each interview lasted for an average of 60 min. Prior to commencement of the data collection a relationship was established with some of the respondents during the research project planning meetings and mobilization phase of data collection.

The quality of data collection was ensured at different steps of the process (piloting and post-piloting revision of tools, collection, transcription, translation, anonymization, digitization/entry into software, coding, and analysis). Mechanisms for quality assurance used included appropriate training (e.g., of transcribers of key concepts/terms used), multiple researchers working on the same data (e.g., coding by at least two researchers), continuous peer-review and peer-support within and between the different partner teams.

Secondary data on facility attendance and utilization from 2012 to 2017 were also collected from the facility Health Management Information System (HMIS) using a standardized pro forma developed for the study. Data was collected from registers stored in the facilities, and from monthly utilization data summaries sent to the local government authorities. The indicators collected include attendance for ANC, facility delivery, child immunization, post-natal care, and family Planning.

### Data Analysis

#### Underpinning Theoretical Framework

To understand how CCT influences utilization of MCH services we drew majorly upon Skinner's (1957) reinforcement theory which focuses on human behavior in our interpretation of the influence of CCT in the uptake of MCH. According to the theory, behavior is a “function of its consequences,” which implies that desirable behavior can be increased through the positive reinforcement technique or rewards. The reinforcers could be financial or non-financial (31, 32). The theory proposes that someone's behavior could be influenced by using reinforcement, punishment, and extinction. The key concept of the theory is “reinforcement,” “punishment,” and “extinction.” Skinner, stated that rewards used to reinforce the desired behavior, punishments are used to avert undesirable behavior while extinction means to terminate a learned behavior. Skinner classified reinforcement into positive and negative reinforcement. The positive reinforcement occurs when the consequence resulting in the behavior one is trying to produce increases the probability that the desired behavior will continue. On the other hand, negative reinforcement is when a negative consequence is withheld if undesired behavior is demonstrated, which will increase the likelihood that the behavior you are seeking out for will continue. Punishment occurs when you enforce a negative consequence to decrease undesirable behavior. While negative reinforcement involves withholding a negative consequence to encourage desirable behavior, punishment is imposing a negative consequence to discourage unwanted behavior. The third concept; extinction trick up Operant Conditioning's sleeve which tries to attempt to terminate a learned behavior by withdrawing the positive reinforcement that stimulated the desired behavior. In this paper, the theory has utility in explaining the use of CCT as a financial reinforcement that stimulates pregnant women's decision to register for and utilize MCH services as well as deliver in health facilities. The extinction concept explains the sudden withdrawal of CCT leading to a reduction in attendance and utilization of MCH services among pregnant women. The desired learned behavior for MCH uptake among pregnant women in the implementing facilities was extinguished although not completely. Similarly, Motivation Crowding Theory reflects on the impact/effect of withdrawal of the monetary incentives in community perceptions of society and motivation. It suggests that discounting monetary incentives of a programme lead to a reduction in intrinsic motivation and, consequently decrease zeal/effort to engage in a task or role (33).

#### Qualitative Data

The study used manual content analysis approach. Audio files were first transcribed in the language of the interview, and then translated to English language, where necessary. Initially two FGD and IDI transcripts with rich information were selected for thorough study and coding. Key themes relating to CCT and its influence on utilization of MCH services were generated and this formed the initial coding scheme. The scheme was then tested on four new transcripts (two FGD and IDI each) and refined into a final coding scheme which was then applied to all the transcripts. After which, related codes were grouped into four broad themes, which were used in interpreting and reporting the findings. The themes identified were: process of payment of CCT; facility attendance and utilization of MCH services; unintended consequences of the provision of CCT and; sustainability of CCT as a strategy for uptake of MCH services.

#### Quantitative Data

The secondary analysis of MCH facility data monthly HMIS data on key MCH indicators were analyzed using SPSS. All were measured as counts per facility across the SURE-P/CCT cluster: (1) total antenatal clinic (ANC) visits (the total number of women that month who visited the PHC for any ANC meeting); (2) total postnatal clinic (PNC) visits, (the total number of women that month who visited the PHC for any PNC meeting) and; (3) number of deliveries taken by a skill birth attendant. Frequencies and simple bar-chart were used for representing the results.

## Results

Based on our analysis of the data, four interrelated broad themes were identified: the process of payment of CCT and its influence on utilization of MCH services; facility attendance and utilization of maternal and child health services; unintended consequences of the provision of CCT and; sustainability of CCT as a strategy for uptake of MCH services. These are presented next.

### Process of Payment of CCT and Its Influence on the Utilization of MCH Services

The payment process of the CCT prompted some pregnant women to comply with facility attendance and complete various stages of the utilization of MCH services to ensure they received the complete payment. This resulted in increased uptake of MCH services (Figure 1). The CCT given to the pregnant women who completed ANC visits, delivered at the facility and PNC visits was made available to the women through different processes, either as a one-off payment or four installment payments. However, CCT facilities paid the eligible women using various mechanisms that were not standardized. A respondent from a CCT facility explained how the payment was made:

“*For the installment mode of payment, women who registered were given one thousand-naira, completion of 4 antenatal visits made the women entitled to two thousand naira, delivery at the facility attracted another payment of one thousand naira while an additional one thousand naira was given for immunizing the child making it a total of five thousand naira. The payment was not made monthly”* (Health worker).

The SURE-P/MCH staff involved in making payments to pregnant women visited the facilities a minimum of twice per year. The visits of the women were documented and the facility would informed the women the day of payment from SURE-P/MCH staff so they will come with their registration cards to the facility for payments. The card will serve as proof of attendance to them in completing the different steps of the process and they will be paid accordingly for one to four visits.

According to the health workers, paying a pregnant woman who had registered and completed her antenatal visits a sum of 3,000 naira motivated the women to utilize the facility and ensured that they fulfilled every requirement that will enable them to collect the complete money.

Pregnant women who registered and completed their antenatal visits but did not deliver at the facility were only paid for what they were due for. This made other women strive to complete the whole process in that way more people came to the facility and continued to attend. From another CCT facility, in the words of one of our respondents:

“*The women know that they won't be paid completely if they don't have a complete visit; they made every effort to see to it that they had a complete visit for their antenatal and delivery so they could receive their complete money”* (WDC).

When the village health workers were asked if the women who did not receive complete CCT were offended or upset, they stated that “*the person would not like it, but she will understand that the mistake was hers. Some of them understood that it was their fault, some were given 3000N and they understood that the reason was that they didn't complete” (VHW)*. Interestingly, women failing to achieve the desired behavior promoted by the CCT seemed to be blamed for this while contextual social and institutional barriers [facility security (reference our paper), cost, unplanned contingencies intrinsic in delivery] were somehow taken for granted in these perceptions.

On a practical level the installment payment (when paid on time) made to the pregnant women was a source of financial help to them to solve the problem transportation to the facility and this motivated them to attend and complete their antenatal visits as stated by family members of the facility users at a CCT implemented facility. “*It helped women very well. It helped them to continue their antenatal. It equally helped a woman whose husband is not financially buoyant. The woman will use that money for transportation.”*

### Facility Attendance and Utilization of Maternal and Child Health Services

**Figure 2:**
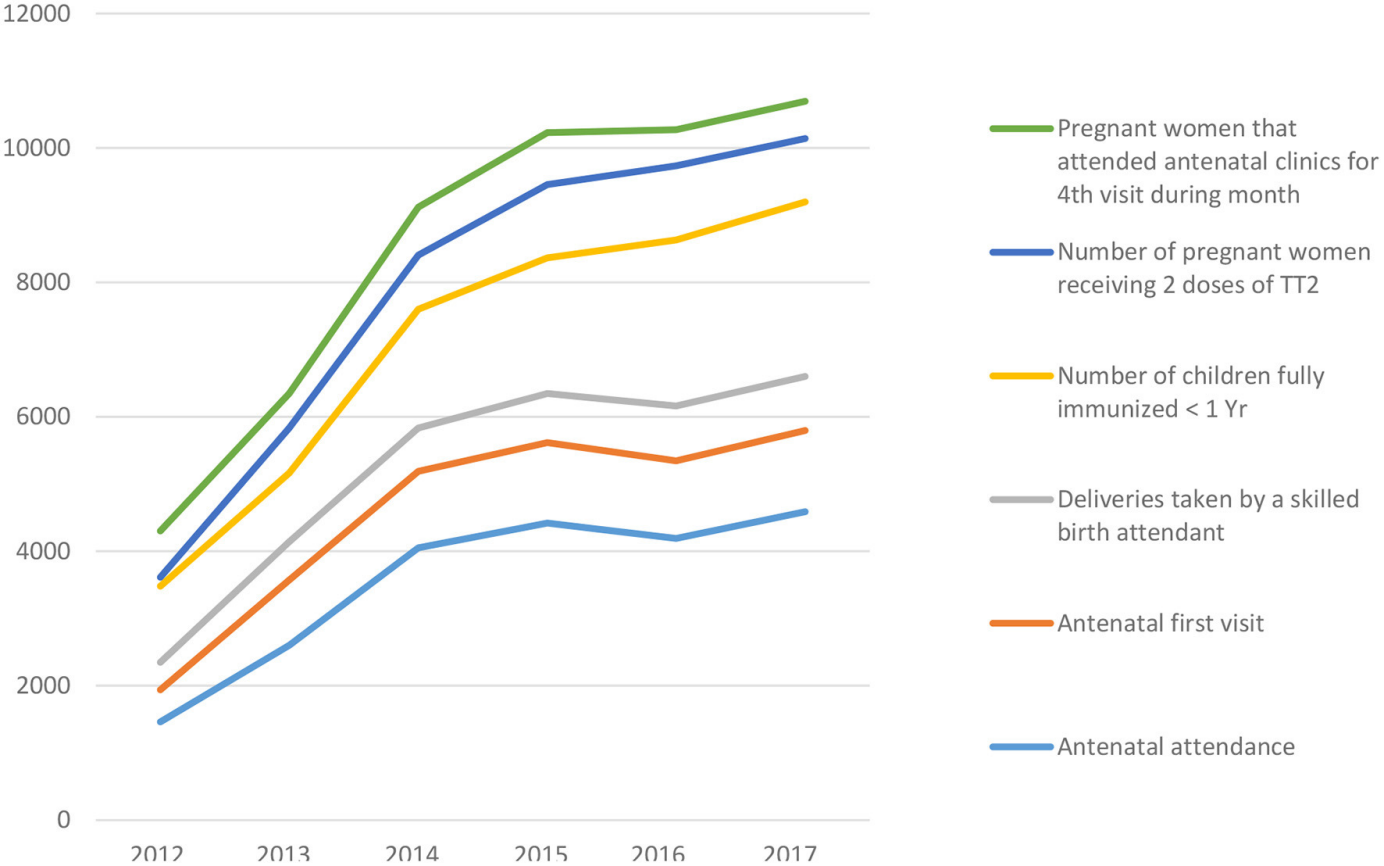
Trend of MCH service uptake pre, during and post-SURE P/MCH intervention among pregnant women between 2012 and 2017 in Anambra state, Nigeria.

Findings from our quantitative data showed a trend of rising utilization of MCH services from 2012 to 2015 in the CCT cluster (Figure 2). There was a steady rise in trend across all the 6 MCH indicators: antenatal attendance, antenatal first visit, antenatal 4th visit, delivery by a skilled birth attendant, pregnant women receiving 2nd dose of tetanus toxoid and the number of children fully immunized at < I year of age.

The findings from qualitative study shows that providing pregnant women with CCT motivated them to attend health facilities with the resultant improved utilization of MCH services (antenatal, delivery, postnatal, and immunization). A health worker from one of the CCT facilities alluded to the fact that there was marked increase in the utilization of facilities, she said “*that going back to the records before SURE-P, the most they had was about 2 deliveries monthly and majority of the time there was no delivery but during SURE-P/CCT, monthly neonatal deliveries rose to 12/ month.”*

A midwife from another CCT facility also buttressed the increase in the number of facility deliveries*; “And when they brought the CCT, we started having many clients. Before we left, our average monthly delivery rose to thirty. So, the CCT attracted them to come.”* A village health worker described it as overcrowding of the facility, and she stated that it was because of the CCT. Other cadres of health workers interviewed in CCT facilities corroborated with the previous workers and their statement was as follows; “*The SURE-P people tried and with that CCT it made the pregnant mothers be coming here” (Facility Manager)*.

CCT motivated some pregnant women to use the facility to the extent that women who would not normally access care at the facility because they do not attach importance to the use of healthcare institutions for pregnancy and delivery started using the facility. Although some other services such as free drugs, delivery, treatment and mama kits accessibility motivated them.

“*There were some women that were used to delivering at home, but because of the CCT, some of them came to the health center for delivery. But if they were not given that CCT, they would deliver at home to avoid paying hospital bills. “The money they received through the programme motivated them to come to the facility and again they had better access to treatments and other drugs needed for antenatal and delivery. This brought about the progress of the health center as more people started to utilize their services” (VHW)*.

Family members of service users and service users also added to the evidence that there was increased utilization of the health facility during the SURE-P/MCH programme, indigenes and non-indigenes alike accessed the facility because of CCT and they were happy with the program.me In their own words, “*the money given to pregnant women helped to draw many women to the hospital,” “it was during that time (SURE-P /CCT) that many people started coming. If you come here at that time, you will see a lot of pregnant women.”*

### Unintended Consequences of the Provision of CCT

Healthcare staff mentioned that some of the women seem to perceive the CCT as an opportunity to collect money from the government and it seem that some were willing to get pregnant in quick succession so as to collect the CCT yet again from the government. The health workers had to counsel them again on the importance of child spacing.

“*. Before long, you will see the person again. We also advise them to watch how they give birth so that they will be able to give birth to the number of children they can raise and not be carried away by free things”. Some of the women interviewed were asking if SURE-P was coming back so they could go and get pregnant again” (VHW)*.

The money was not always used to pay for direct maternal and child health commodities. For example, some women started businesses with the money while others used it to solve other personal problems. In the words of a village health worker in one of the CCT facilities,

“*The money that they received was seriously empowerment to them. I have seen a woman who came here and collected hers, since she collected it completely at once she used it to start selling something immediately, which she is now using to feed the child. There is another woman who collected hers and used it to buy pesticides for her crops. From there, she empowered herself”. Also, a family member of service users said that “the money-motivated them (Services user) to keep coming to this health center. Even if you are a millionaire no money is small money. At times, if you are given* ₦*1,000.00 you can use it to solve a big problem” (VHW)*.

### Sustainability of CCT as a Strategy for Uptake of MCH Services

The decline in uptake of MCH services following withdrawal of the programme is shown in data utilization patterns (Figure 2) where there was an obvious decline across all the MCH service indicators as presented by a dip on the line graph in 2016 immediately after the programme was terminated.

In the word of a village health worker, “*That is not true there is a significant difference in the number of people that are utilizing it now and those that were utilizing it then. If 100 people were visiting the facility during SURE-P, now there are just 20 persons that are utilizing. Do you not see that the difference is much?”, Family members of service users in another CCT facility confirmed the statement of the health worker. They said that during SURE-P the facilities were usually overcrowded but now post sure the place had become empty with very scanty attendance*.”

The group of women who would not ordinarily use the facility that did because of the CCT also stopped coming. “*After the SURE-P MCH programme, some people were still coming but people that were not used to coming stopped coming” (VHW)*. Another health worker said she noticed that since SURE-P stopped people no longer came to the facility as before. And one of them gave the reason being that it was because CCT was stopped. “*When you tell them to come now, they will tell you they won't that they don't give money anymore* (referring to the CCT payment)” *(VHW)*. Some clients who were coming from other villages that were not walking distances to the facility also stopped attending because they could not afford the transportation fare and had no hope of collecting CCT.

The withdrawal of the conditional cash transfer and the SURE-P/MCG programme also led some to distrust in the health system and subsequent reduction in utilization of MCH services. Consequently, some women began to visit other service providers, including TBAs. In the word of a WDC member, “*The problem of inconsistencies in government policy is discouraging to some people. I once met a woman whom I talked to about coming to the health center and she reminded me that her daughter gave birth and was then not among those that were paid because the programme has seized to operate. She said I should leave her alone those things about government are not trustworthy” (WDC)*.

However, despite the withdrawal of the programme and its financial incentives, some service users still have confidence in health system and maintained unwavering trust in health providers due to their satisfaction and the level of interpersonal trust previously built on the health providers.

## Discussion

This study highlights three key issues the positive effect of CCT; the unintended consequences of CCT; and the sustainability of CCT in MCH services uptake. The positive effects relate to how CCT improved uptake of MCH services directly as a motivating factor and indirectly due to the method of payment of CCT. The unintended consequences of CCT relate to the effect of CCT that although was beneficial to the recipient but not a goal for implementing CCT, and thirdly, the failure of the programme to sustain its goal in the long term.

Our study revealed that implementing CCT contributed to increased uptake and utilization of MCH services in the study facilities. This could be attributed to the anticipated reinforcer/reward (CCT) as Skinner postulated in his reinforcement theory which states that behavior is a function of its consequences, on which our study hinged on (32). The implication is that CCT was a good motivating factor for some women. It undoubtedly took care of some financial barriers which are a major reason for poor health-seeking behavior especially among rural residents in SSA (34–36) as well as the opportunity cost of being away from income-generating activities during a healthcare-seeking time (3, 37). Since the incentive was paid post-behavior, those motivated by the CCT had to have some prior resources that allowed them to perform the desired behavior (e.g., pay for transport) before collecting the money weeks or months later.

More so, our findings are suggestive of temporary reduction of home and traditional birth deliveries in the study communities since pregnant women who would ordinarily not use health facilities started doing so. This finding conforms to the assertion that cash incentives can be useful in promoting positive health-seeking behaviors among women in rural communities (38). Our finding is in consistence with a systematic review on the impact of CCT on Maternal and Newborn Health in Kenya, Malawi, India, Nepal, Uruguay, Cambodia, and South Africa which showed a significant increase in the utilization of different MCH service (prenatal monitoring, ANCs, facility deliveries and, immunization) (22, 28, 39) among the target population. A similar study in Nigeria corroborated with our study showing a significant increase in more women attending four or more ANC visits, facility birth, and receiving two or more tetanus toxoid doses during pregnancy (28, 40). In the context of other healthcare preventive and curative services, CCT had been previously reported to improve: HIV testing, care, and prevention (13, 41); completion of tuberculosis treatment regimens (42, 43); adherence to the hepatitis B vaccine (44) and; nutritional uptake (45).

The payment process of CCT instilled the desired behavior among pregnant women who were determined to complete different stages of the conditions attached to payment of the monetary incentives. This suggests that women's continual efforts to meet the condition for CCT were stimulated leading to improved MCH uptake which would have been lower if the payment was one-off. Our finding has important public health implications in regions where uptake of MCH services is low.

We found that the CCT which was intended to motivate women to use health facility resulted in a reduction in birth spacing intervals. The women receiving CCT saw it as an opportunity to get money from the government, so, many of them were getting pregnant quickly to benefit from the financial incentive. The long term effect of such programmme could be outrageous increase in population particularly among CU5. Our finding is in line with other studies that show that conditionality attached to the CCT undermined the programme's objective. For instance, a financial incentive programme intended to increase demand for public maternity services was observed to increase pregnancy rates (18, 46). Another study reported in Brazil, where CCT was designed to improve children's nutrition outcomes, but, led to some parents under-investing in their children's health, which resulted to reduction in their weight due to the erroneous parents perception that CCT and other programme's benefits would be discontinued if the children grow well (47). Additionally, we found that CCT was a financial aid for establishing petty businesses and solving personal problems outside what it was intended for. Our study agrees with previous studies which averred that CCT was used in addressing poverty as beneficiaries indicated that they utilized the money in establishing businesses (40, 48). This is not a surprise as 40.1% of the total population of Nigeria were categorized as being poor and has actual per capita expenditure less than N137,430($381) per year (49).

As evident in our study implementing CCT as a behavioral change tool for MCH uptake was not a sustainable strategy as some women were demotivated from using health facilities following its withdrawal, thus decline in MCH services attendance. It was envisaged that with CCT, overtime, the women would come to understand the importance of accessing health care during pregnancy and delivery given that they were taught all the time and they confessed to their observed reduction in maternal and child mortality. Despite that, as soon as the programme ended and over time they realized the money was not going to be paid anymore, most of them withdrew from utilizing MCH services in the facility. The majority of village health workers observed a reduction in the number of clients. This was demonstrated in the dip curve as shown in figure although the curve did eventually continue declining afterward probably due to other MCH programmes (e.g., Saving One Million Lives) that commenced soon after SURE-P/MCH programme was withdrawn by the new federal government. This is suggestive that for CCT programmes to be sustained and possibly scaled-up, it is largely dependent on political will and support. This is evident in the literature (11, 23, 26) suggesting that the use of financial incentives to advance a population's health-seeking behavior, at times, do not last long because it does not impact intrinsic motivation and there the life span seem dependent on political support available to them (10). The inability of financial incentives to sustain long-term health improvements have been often reported (10) and the authors equally noted that the sustainability of financial incentives programmes is dependent on efforts of policy champions in donor and governmental organizations to ensure political and financial support and on whether it is subject to specific political interests, and recognizing this has proven helpful for programmes in other LMIC countries such as Kenya and Uganda.

It is important that policymakers and government should reflect on and build-in a sustainable structure in policy designs to mitigate sudden programme withdrawal and its subsequent effects on service users and the health system at large. Exploring other forms of incentives which could provide long-term benefits to beneficiaries while also putting into consideration measures to avert or control negative unintended consequences of such a sustainable strategy.

### Strengths and Limitations of the Study

A major strength of this study is that it explored the views of different actors, who were either directly or indirectly involved with the planning and/or implementation of the programme. The cross-cutting response supplied by the respondents provided an in-depth overview of how CCT influenced MCH uptake in Anambra State. More so, using mixed methods; qualitative and quantitative (pre and post service utilization data) contributed largely to the richness of the study because it enabled complementarities and validation findings through triangulation.

Our study has some limitations. First, results of our sample may not be generalizable to others states/Nigeria, since we collected data from just 1 state out of 9 states that implemented SURE-P/MCH+CCT. However, principles highlighted in this study may be transferable to similar contexts. Secondly, we did not explore the influence of supply side factors on the MCH uptake, which may have also contributed to increased uptake of MCH services in the study communities. Furthermore, although ANC, health facility delivery and PNC utilization increased during the programme implementation, it remains unknown whether CCT has a significant effect on pregnancy outcomes, which this study did not measure, this highlight an area for further study.

## Conclusion

In this study, it has been established that CCT incentivized pregnant women leading to increased uptake of ANC, facility delivery, and PNC which had helped in improving temporary access to these MCH services as more women were able to use health facilities better than before the programme in Anambra State, Nigeria. Ultimately, in combination with other measures, it removed financial barriers to accessing MCH services. The programme equally had some unintended outcomes which include assisting beneficiaries financially in setting-up businesses to advance their standard of living; reduction in childbirth spacing and trust in the health system. However, withdrawal of the CCT led to a decline in uptake of MCH services in the study facilities. CCT as a social intervention for the uptake of MCH services is a sustainable strategy. The findings will be useful to policymakers and the government in designing and implementing health programmes to enhance access to health services.

## Data Availability Statement

The datasets presented in this study can be found in online repositories. The names of the repository/repositories and accession number(s) can be found below: University of Leeds SAN (Storage Area Network) repository, http://library.leeds.ac.uk/info/422/policies/189/university_of_leeds_research_data_management_policy/1.

## Ethics Statement

The studies involving human participants were reviewed and approved by Ethical approvals were obtained from the Faculty of Medicine and Health at the University of Leeds, United Kingdom (ref: SoMREC/14/097), and the Health Research Ethics Committee of the University of Nigeria Teaching Hospital, Ituku-Ozalla, Enugu (Ref: NHREC/05/02/20088-FWA00002458-IRB00002323). The patients/participants provided their written informed consent to participate in this study.

## Consent for Publication

Informed consent for publishing anonymised data was obtained from all study participants prior to the interviews.

## Author Contributions

AM, TE, OO, BU, and TM conceptualized the study. UE, CO, PO, UA, and EE participated in data collection and involved in data analysis. UE, CO, PO, UA, EE, and BE prepared the first draft of the manuscript. All authors reviewed and approved the final version of the manuscript.

## Conflict of Interest

The authors declare that the research was conducted in the absence of any commercial or financial relationships that could be construed as a potential conflict of interest.

## Abbreviations

CCT: conditional cash transfer
MCH: maternal and child health
CU5: children under-five
SURE-P: Subsidy Reinvestment and Empowerment Programme
SURE-P/MCH: Subsidy Reinvestment and Empowerment Programme, Maternal and Child health
CHEW: Community health extension worker
PHC: Primary health centre
VHW: village health worker
WDC: ward development committee
ANC: ante-natal care
HMIS: health management information system.
